# Icariin inhibits the expression of IL-1β, IL-6 and TNF-α induced by OGD/R through the IRE1/XBP1s pathway in microglia

**DOI:** 10.1080/13880209.2021.1991959

**Published:** 2021-10-28

**Authors:** Zhen-Tao Mo, Jie Zheng, Yu-ling Liao

**Affiliations:** Department of Pharmacology of Zhuhai Campus, Zunyi Medical University, Zhuhai, China

**Keywords:** Microglia, oxygen-glucose deprivation, IRE1, inflammation

## Abstract

**Context:**

Icariin (ICA), a flavonol glycoside extracted from *Epimedium brevicornum* Maxim (Berberidaceae), has been proven to inhibit inflammatory response in ischaemic rats in our laboratory's previous work. However, its underlying mechanism is still unclear.

**Objective:**

This study investigates the effects of ICA on endoplasmic reticulum (ER) stress mediated inflammation induced by cerebral ischaemia–reperfusion (I/R) injury *in vitro*.

**Materials and methods:**

The primary cultured microglia were treated with oxygen-glucose deprivation (OGD) for 2 h followed by a 24 h reoxygenation. ICA (0.37, 0.74 and 1.48 μmol/L) administration was performed 1 h prior OGD and acting through 2 h OGD. The control group was cultured in normal conditions. At 24 h after reoxygenation, the expression of IRE1α, XBP1u, XBP1s, NLRP3 and caspase-1 was detected by western blotting (WB) and quantitative real-time (qRT) PCR; the expression of p-IRE1α was examined by WB; the expression of IL-1β, IL-6 and TNF-α was measured by WB and enzyme-linked immunosorbent assay (ELISA).

**Results:**

ICA (0.37, 0.74 and 1.48 μmol/L) reduced the ratio of p-IRE1α/IRE1α, the mRNA level of IRE1α, the expression of XBP1u, XBP1s, NLRP3, caspase-1 at both the mRNA and protein level expression of IL-1β, IL-6 and TNF-α in OGD/R injured microglia. Overexpression of IRE1 significantly reversed the effects of ICA.

**Discussion and conclusions:**

These results suggested that ICA might decrease the expression of IL-1β, IL-6 and TNF-α by inhibiting IRE1/XBP1s pathway. The anti-inflammatory effect of ICA may provide a potential therapeutic strategy for the treatment of brain injury after stroke.

## Introduction

Complex pathophysiological changes, including inflammatory injury, energy metabolism disorder, increased excitatory amino acid, intracellular Ca^2+^ overload and free radical damage are involved in the process of cerebral ischaemia–reperfusion (I/R) (Mahura 2003). It was reported that inflammatory mediators were induced by cerebral I/R models *in vivo* and *in vitro* (Wu et al. 2017). Inflammation is one of the main causes of neuronal death, which runs through the pathological development after I/R (Su et al. 2017). Therefore, reducing inflammatory injury may be one of the important strategies to alleviate I/R injury. Recently, it is reported that endoplasmic reticulum (ER) stress is involved in the regulation of inflammatory response (He et al. 2019). ER stress is a state in which ischaemia, hypoxia, oxidative stress and other factors lead to the accumulation of misfolded and unfolded proteins in ER, resulting in the disorder of cell physiological function. When ER stress occurs, ER initiates unfolded protein response (UPR), which helps cells restore homeostasis by inhibiting protein synthesis, promoting degradation of misfolded proteins and folding of unfolded proteins (Krebs et al. 2015). However, excessive or persistent ER stress can induce inflammation (He et al. 2019). IRE1α-XBP1 signalling pathway is one of the highly conserved ER stress basic pathways. IRE1α is a type I transmembrane protein on the ER, and has both kinase and ribonucleic acid endonuclease activities. Its amino terminal in the ER can sense unfolded proteins and misfolded proteins and bind to them. This process activates ribonucleic acid endonucleases at the carboxyl end and cleaves about 26 bp-long introns in the cytoplasm of X-box binding protein 1 (XBP1) to form spliced XBP1 (XBP1s, activated type). This process activates ribonucleic acid endonucleases at the carboxyl end and shears the 26 BP content of XBP1 in the cytoplasm. XBP1s, which enters the nucleus and binds to UPR elements on DNA, enhances the expression of ER stress-related proteins (Yuan et al. 2014). XBP1s can not only promote inflammation by mediating activation of NF-κB, but also activate NLRP3-mediated inflammatory response (Tam et al. 2012). XBP1 activates inflammatory bodies of NLRP3, which converts precursor caspase-1 into enzymatic caspase-1, and then the latter catalyses precursor IL-1β into active IL-1β which is secreted outside cells (Yue et al. 2016).

*Epimedium brevicornum* Maxim (Berberidaceae) is a traditional Chinese herbal medicine. Several clinical case reports have also shown that *Epimedium*-containing prescriptions can significantly improve the ability of daily living of stroke patients in recovery period, and reduce the degree of neurological deficits (Deng 2008; Wen and Zeng 2017). Pharmacological studies support its clinical application with the results of the neuroprotective effects of icariin (ICA) on cerebral ischaemic injury (Zhu et al. 2010; Liu et al. 2018). ICA is one of the major effective flavonoid glycosides extracted from the leaves and stems of *Epimedium*. A previous work of our laboratory showed that ICA could reduce brain oedema, cerebral infarction area and levels of inflammatory mediators including IL-1β and TGF-β_1_ in I/R rats (Xiong et al. 2016). ICA could also reduce the secretion of heat shock protein and inflammatory cytokines such as IL-1β, IL-6 and TNF-α in oxygen-glucose deprived cells *in vitro* (Mo et al. 2015, 2017). The results of these experiments confirmed that ICA can inhibit inflammation *in vivo* and *in vitro*. However, how ICA regulates this process remains unclear. In this study, we investigate the effect of ICA on the NLRP3/caspase-1/IL-1β inflammatory signal axis mediated by IRE1α-XBP1 signalling pathway *in vitro*.

## Materials and methods

### ICA preparation

The purity of ICA (Chengdu Alfa Biotechnology Co., Ltd., Chengdu, China) was more than 98% (by high-performance liquid chromatography). It was dissolved in Earle’s balanced salt solution or full culture medium. The composition of Earle’s balanced salt solution and Full culture medium was previously described (Mo et al. 2017, 2020).

### Primary microglia culture

The isolation, purification and culture of primary microglia were described before (Mo et al. 2020). Briefly, microglia were seeded in six-well plates with poly-l-lysine at a density of 2 × 10^5^ cells/well. Half of the maintenance medium was replaced every three days. All experiments were approved and performed according to the guidelines of the Ethics Committee of Ethics Committee of Zunyi Medical University.

### Primary microglia purity identification

The purity identification of primary microglia was described before (Mo et al. 2020). Briefly, the cells were incubated with CD11b primary antibody (Santa Cruz Biotechnology, Inc., Santa Cruz, CA; 1:50 diluted in PBS). Then, CY3 secondary antibody (Aspen Biotech, Shanghai, China; 1:50 diluted in PBS) and DAPI (Aspen Biotech, Shanghai, China) were used. The image was observed under a fluorescence microscope.

### Lentiviral transfection and collection

Lentiviral transfection and collection were described before (Mo et al. 2020). HEK293T cells (4.8 × 10^6^) were seeded in a 10 cm diameter dish for 24 h. After that, the cells were transferred into the DMEM complete medium with sodium pyruvate. It was noticed that the culture medium was substituted for the antibiotic-free medium 2 h before transfection. The mixture of plasmid DNA (5 μg of expressing plasmid, 9 μg of packaging plasmids), Lipofectamine 2000 (68 μL) Opti-MEM medium (3 mL) was incubated for 30 min. Then, the mixture was added to culture dishes and incubated in incubator. The medium was centrifuged and filtered (0.45 μm) after 72 h transfection. Subsequently, the supernatants were precipitated and centrifuged by lentivirus concentrate (ELK Biotech, Wuhan, China). The precipitates were dissolved in HBSS solution and stored at −80 °C until use. Titres were 2 × 10^8^ transduction U/mL.

### IRE1 overexpression in microglia

The expressing plasmid Phs-AVC-IRE1 (GenScript Biotech, Nanjing, China) and packaging plasmid pLP1, pLP2, pLP/VSVG (Invitrogen, Carlsbad, CA) were co-transfected in 293T cells. The culture medium containing the virus was collected as mentioned in section ‘Lentiviral transfection and collection’. Neonatal rat microglia were plated in six-well plates with complete medium containing the virus for 1 d. The next day, microglia were infected with IRE1 overexpressed lentivirus and incubated for 12 h. Microglia were gathered 72 h after transfection, and the transduction rate was about 90%.

### OGD/R and drugs

The primary cultured microglia were divided into the following eight groups: normal control group, oxygen-glucose deprivation and reoxygenation (OGD/R) group, ICA low dose group (0.37 μmol/L), ICA medium dose group (0.74 μmol/L), ICA high dose group (1.48 μmol/L), empty vector group, IRE1 alpha overexpression group and ICA high dose + IRE1α overexpression group. Cells of each group were performed as described previously (Mo et al. 2020). Briefly, microglia were subjected to OGD for 2 h followed by a 24 h reoxygenation. ICA (0.37, 0.74 and 1.48 μmol/L) administration was performed 1 h prior OGD and acting through 2 h OGD.

### Western blotting (WB)

The extraction of proteins was described previously (Mo et al. 2020). Membranes were probed with primary antibodies against IRE1α (1:500, Santa Cruz Biotechnology, Inc., Santa Cruz, CA), p-IRE1α (1:1000, Invitrogen, Carlsbad, CA), XBP1 (1:1000, Santa Cruz Biotechnology, Inc., Santa Cruz, CA), NLRP3 (1:500, Abcam, Cambridge, UK), caspase-1 (1:500, Santa Cruz Biotechnology, Inc., Santa Cruz, CA), IL-1β (1:1000, Santa Cruz Biotechnology, Inc., Santa Cruz, CA), IL-6 (1:1000, Santa Cruz Biotechnology, Inc., Santa Cruz, CA), TNF-α (1:1000, Santa Cruz Biotechnology, Inc., Santa Cruz, CA) and GAPDH antibodies (1:10,000, Abcam, Cambridge, UK). Then, HRP-conjugated goat anti-rabbit/mouse IgG antibody (1:10,000, Aspen Biotech, Shanghai, China) was used. The bands were visualized with the ECL kit (Aspen Biotech, Shanghai, China) and the intensity of bands was analysed with Band-Scan software.

### Quantitative real-time PCR (qRT-PCR)

Total RNA was extracted with Trizol reagent (ELK Biotech, Wuhan, China) according to the manufacturer’s protocol. RNA was reversed to cDNA by M-MLV Reverse Transcriptase (ELK Biotech, Wuhan, China). QRT-PCR was operated using QuFast SYBR Green PCR Master Mix kit (Life Technologies, Grand Island, NY). GAPDH was used as an internal reference. The sequences of primers were as follows:

IRE1α (F: 5′-GGAGACCCTACGCTATTTGACCT-3′, R: 5′-CTTCGAGCAAAGGAAGAGTGCT-3′), XBP1U (F: 5′-TTGTCACCTCCCCAGAACATC-3′, R: 5′-TTCCTCCAGATTAGCAGACTCTG-3′), XBP1S (F: 5′-AACTCATGGGCTTGTGATTGAG-3′, R: 5′-CAGATTAGCAGACTCTGGGGA-3′), NLRP3 (F: 5′-AACCAGAGCCTCACTGAACTGG-3′, R: 5′-AGAGCAGATGCTTCAGTCCCAC-3′), caspase-1 (F: 5′-GCCTGGTCTTGTGACTTGGAG-3′, R: 5′-TCCTGGGAAGAGGTAGAAACG-3′) and GAPDH (F:5′-CGCTAACATCAAATGGGGTG-3′, R: 5′-TTGCTGACAATCTTGAGGGAG-3′). Relative expression was calculated with a comparative ΔΔCT method.

### Enzyme-linked immunosorbent assay (ELISA)

IL-1β, IL-6 and TNF-α in the supernatant fluid were analysed by IL-1β, IL-6 and TNF-α ELISA kits (ELK Biotech, Wuhan, China). The assay was performed according to the manufacturer’s protocol (Mo et al. 2020).

### Statistical analysis

All results are expressed as mean ± standard deviation (SD). Statistical significance was analysed with one-way factor analysis of variance (ANOVA) followed by least significant difference (LSD) tests (SPSS 16.0, Chicago, IL). Values of *p* < 0.05 and *p* < 0.01 were regarded as statistically significant.

## Results

### Primary microglia purity identification

**Figure 1.**
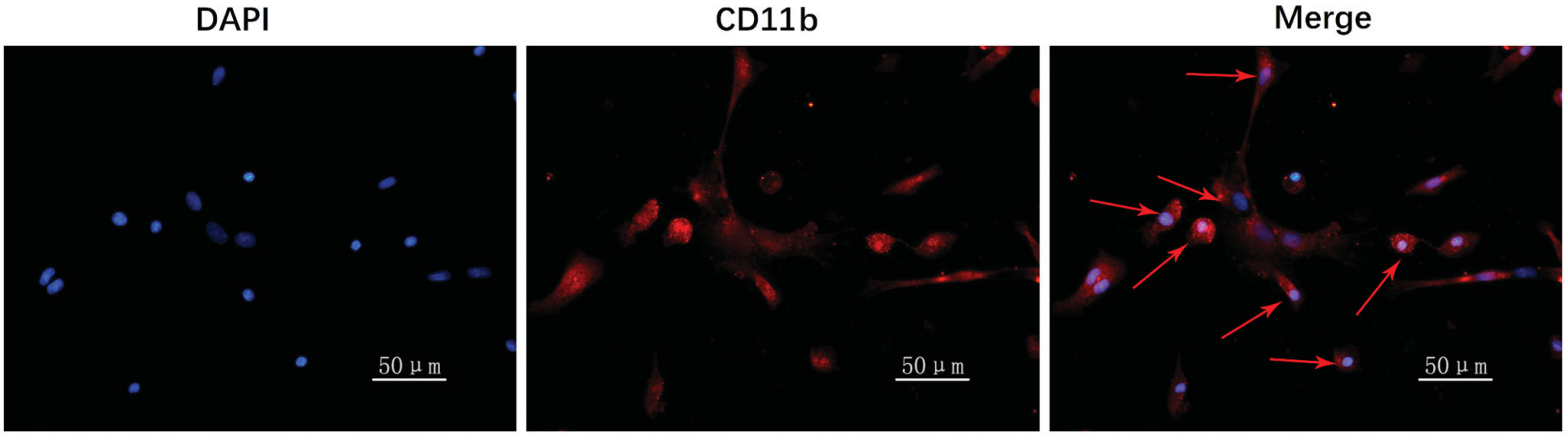
Primary microglia purity was identified by immunofluorescence. Blue signal indicates nuclear staining with DAPI; red signal represents CD11b staining with CY3; arrows represent microglia with typical morphology.

Prior to the subsequent experiments, the purity of primary microglia was identified by immunofluorescence using the specific marker CD11b. The purity of primary cultured microglia was more than 90% (Figure 1).

### Transduction rate of lentiviral plasmid in microglia

**Figure 2.**
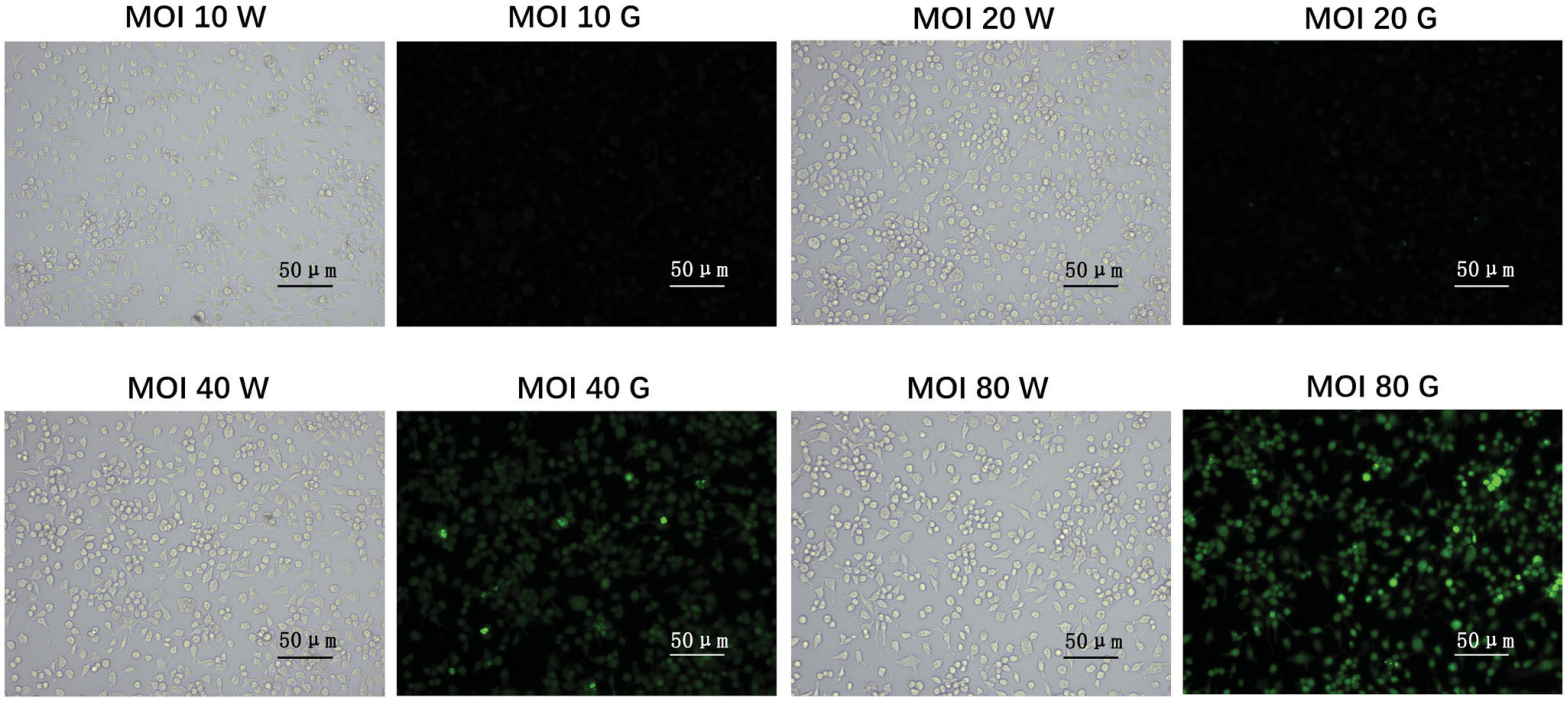
Transduction rate of lentiviral plasmid in microglia. Neonatal rat microglia were plated in six-well plates with complete medium 24 h prior infection. Next day, microglia were infected with IRE1 overexpressed lentivirus at different multiplicity of infection (MOI) value (10–80) and incubated for 12 h. Microglia were gathered 72 h after transfection, and transduction efficiency was measured with an inverted fluorescence microscopy (Olympus, Tokyo, Japan). Green signal represents infected cells. Transduction rate was approximately 90% at an MOI of 80. W: observation under white light; G: observation under green light.

The transduction efficiency of lentiviral plasmid was measured with an inverted fluorescence microscopy. Transduction rate of lentiviral plasmid was about 90% (Figure 2).

### ICA suppressed IRE1α/XBP1 inflammatory signal axis induced by OGD/R

**Figure 3.**
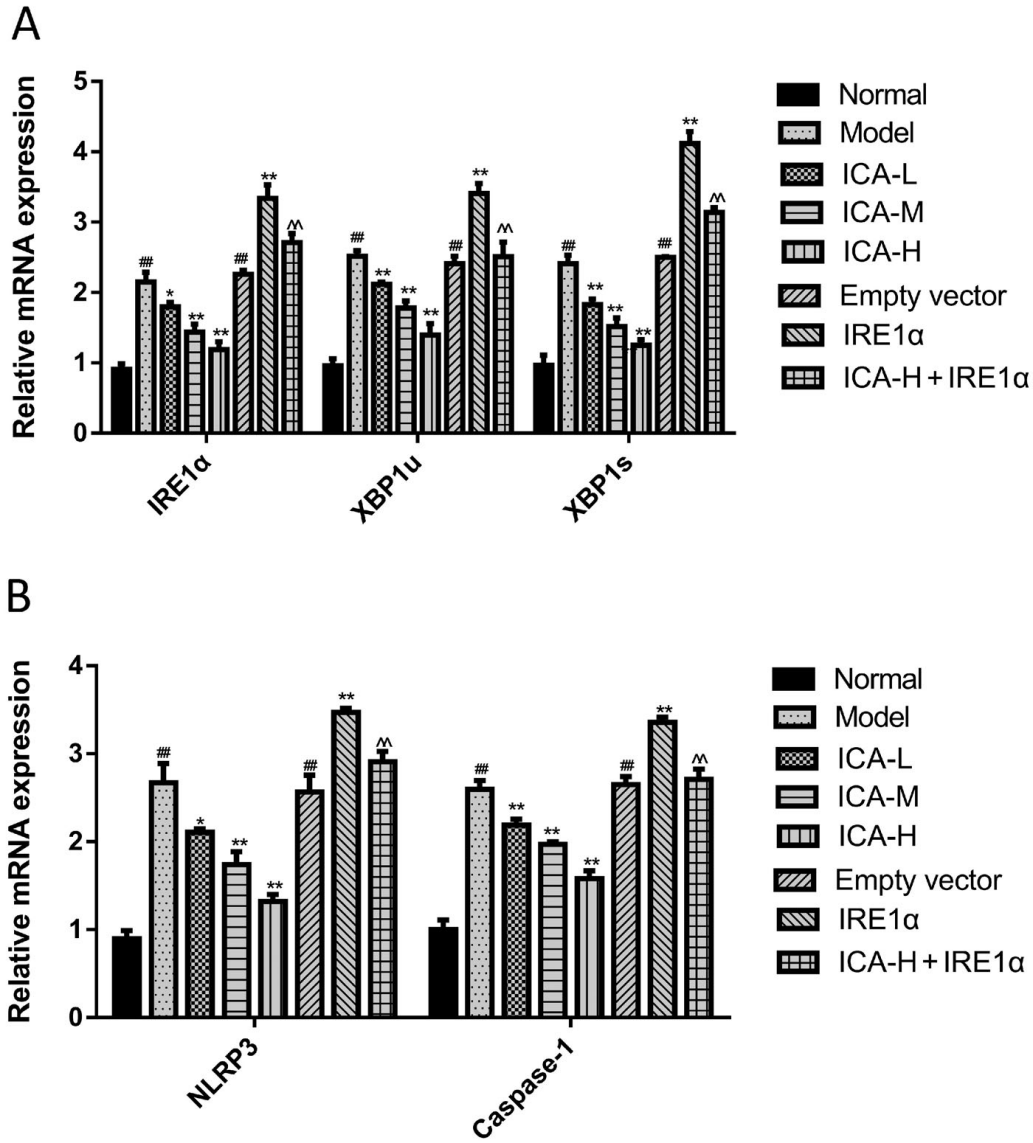
ICA reduced the mRNA expression of IRE1α/XBP1 inflammatory signal axis induced by OGD/R. Microglia were subjected to 2 h of OGD followed by 24 h reoxygenation. ICA (0.37, 0.74 and 1.48 μmol/L) administration was performed 1 h prior OGD and maintained 2 h throughout OGD. IRE1α, XBP1u, XBP1s, NLRP3 and caspase-1 mRNA expression was assayed by qRT-PCR. Data are expressed as mean ± SD of three independent experiments. ^##^*p*< 0.01 vs. normal control group; *p< 0.05, **p< 0.01 vs. OGD/R group; ^^^^*p*< 0.01 vs. ICA-H group. ICA-L: 0.37 μmol/L ICA, ICA-M: 0.74 μmol/L ICA and ICA-H: 1.48 μmol/L ICA.

**Figure 4.**
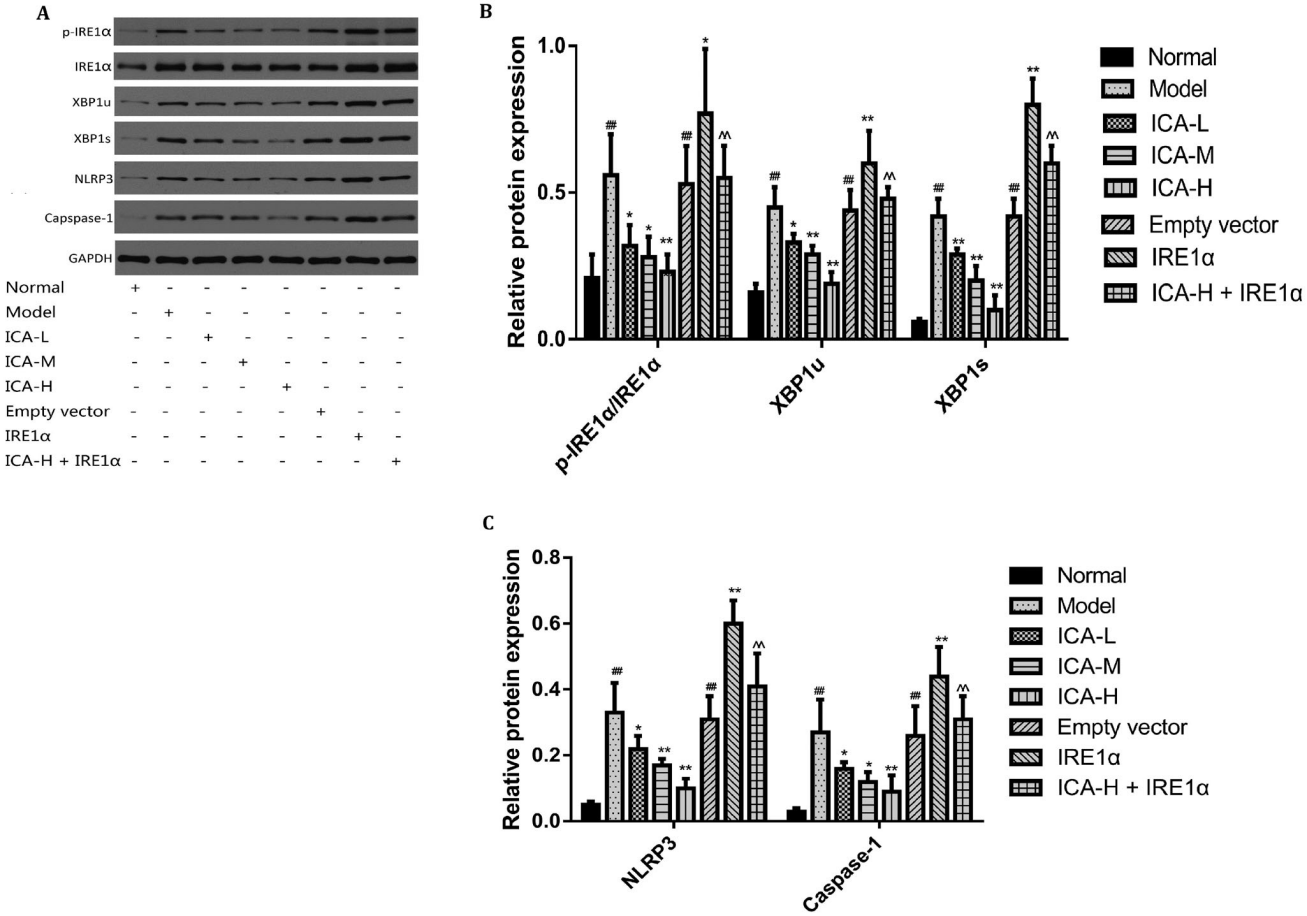
ICA suppressed the protein expression of IRE1α/XBP1 inflammatory signal axis induced by OGD/R. Microglia were suffered from 2 h of OGD followed by 24 h reoxygenation. ICA (0.37, 0.74 and 1.48 μmol/L) administration was performed 1 h before OGD and acting through 2 h OGD. IRE1α, p-IRE1α, XBP1u, XBP1s, NLRP3 and caspase-1 protein expression was examined by WB. Data are expressed as mean ± SD of three independent experiments. ^##^*p*< 0.01 vs. normal control group; *p< 0.05, **p< 0.01 vs. OGD/R group; ^^^^*p*< 0.01 vs. ICA-H group. ICA-L: 0.37 μmol/L ICA, ICA-M: 0.74 μmol/L ICA and ICA-H: 1.48 μmol/L ICA.

XBP1s could activate NLRP3-mediated inflammatory signalling pathway (Yue et al. 2016). To test this possibility, microglia were suffered to 2 h of OGD followed by 24 h reoxygenation. ICA administration was performed 1 h prior OGD and acting through 2 h OGD. IRE1 overexpression group was set up to explore the molecular mechanisms of IRE1α/XBP1 regulating inflammatory pathway. We found that the mRNA levels of IRE1α, XBP1u, XBP1s, NLRP3, caspase-1 and the protein expression of XBP1u, XBP1s, NLRP3, caspase-1 as well as the ratio of p-IRE1α/IRE1α were significantly increased by OGD/R treatment (*p* < 0.01). However, ICA treatment reduced the levels of the above factors significantly (*p* < 0.05 and *p* < 0.01). Overexpression of IRE1 significantly reversed the effects of ICA (*p* < 0.01) (Figures 3 and 4).

ICA inhibited the expression and release of IL-1β, IL-6 and TNF-α induced by OGD/R.

**Figure 5.**
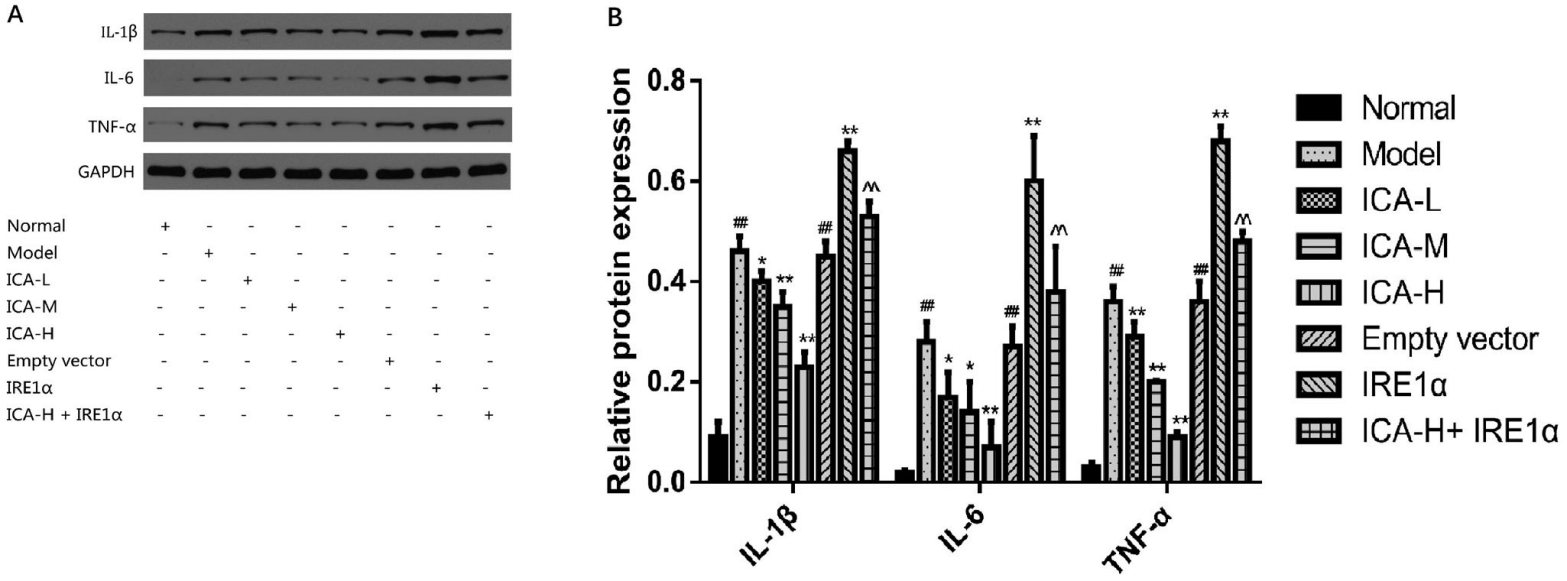
ICA inhibited the expression and release of IL-1β, IL-6 and TNF-α induced by OGD/R. Microglia were suffered to 2 h of OGD followed by 24 h reoxygenation. ICA administration was performed 1 h prior OGD and acting through 2 h OGD. IL-1β, IL-6 and TNF-α protein levels were measured by WB and ELISA. Data are expressed as mean ± SD of three independent experiments. ^##^*p* < 0.01 vs. normal control group. *p< 0.05, **p < 0.01 vs. OGD/R group. ^^^^*p* < 0.01 vs. ICA-H group. ICA-L: 0.37 μmol/L ICA, ICA-M: 0.74 μmol/L ICA and ICA-H: 1.48 μmol/L ICA.

To further examine the effect of ICA on inflammatory response induced by OGD/R in microglia, IL-1β, IL-6 and TNF-α protein levels were measured by WB and ELISA. The results of ELISA were reported in our previous study (Mo et al. 2020). As shown in Figure 5, the results showed that protein levels of IL-1β, IL-6 and TNF-α were markedly increased in OGD/R group (by WB: IL-1β: 0.46 ± 0.03, IL-6: 0.28 ± 0.04, TNF-α: 0.36 ± 0.03; by ELISA: IL-1β: 69.95 ± 6.72 ng/L, IL-6: 163.16 ± 11.68 ng/L, TNF-α: 73.24 ± 6.01 ng/L), compared to normal control group (by WB: IL-1β: 0.09 ± 0.03, IL-6: 0.02 ± 0.00, TNF-α: 0.03 ± 0.01; by ELISA: IL-1β: 25.74 ± 5.72 ng/L, IL-6: 83.19 ± 5.64 ng/L, TNF-α: 18.42 ± 3.74 ng/L; *p* < 0.01). However, ICA decreased IL-1β, IL-6 and TNF-α levels significantly in ICA + OGD/R group (*p* < 0.05 and *p* < 0.01). Overexpression of IRE1 significantly reduced the effects of ICA (*p* < 0.01).

## Discussion

A previous study has proved that ICA can reduce the inflammatory response induced by cerebral I/R (Xiong et al. 2016). Recent studies have shown that ER stress, a pathological condition caused by cerebral I/R, may play a profound role in regulating the inflammatory response (Anuncibay-Soto et al. 2018). Whether ICA can regulate the inflammatory response of cerebral I/R by interfering with ERS is unclear. Microglia, the macrophage-like cells, are the most important immune cells in the brain. Under the pathological conditions of ischaemia, infection and trauma, microglia can rapidly activate and proliferate, migrate to the injured site, and secrete a large number of inflammatory cytokines (Mallard et al. 2019). Therefore, in the present study, we chose primary cultured microglia as the research object.

ER is an important site for intracellular protein synthesis, modification, folding and regulation of calcium storage. When cells are stimulated by ischaemia, hypoxia, oxidative stress and disordered calcium metabolism, accumulation of unfolded and misfolded proteins in ER leads to a failure of ER homeostasis, resulting in ER stress which is activated through three important signalling pathways: PERK, ATF6 and IRE1 (Anuncibay-Soto et al. 2018; Cui et al. 2019). Under mild ER stress conditions, activation of these signalling pathways can reduce the pressure of ER and maintain the stability of intracellular environment by reducing protein synthesis, promoting folding and degradation of unfolded and misfolded proteins (Bravo et al. 2012; Xie et al. 2016). Conversely, under serious ER stress conditions, the stress exceeds the capacity of UPR, and the homeostasis of the ER cannot be restored, so the activation of these signal pathways can induce cellular apoptosis (Bravo et al. 2012; Pinkaew et al. 2017).

However, overactivated IRE1α-XBP1 signalling pathway can also induce inflammatory response (Yue et al. 2016). IRE1α is a type I transmembrane protein on ER, which has both kinase and endoribonuclease activities. Under physiological conditions, IRE1α exists in ER as a monomer. When the cells are stimulated by ischaemia and oxidative stress, the ER lumen of unfolded and misfolded proteins accumulates, which stimulates the dimerization of IRE1α, and the carboxyl terminus of IRE1α is subsequently autophosphorylated, thereby activating its kinase and endoribonuclease activities (Concha et al. 2015). Activated IRE1α can phosphorylate and activate its downstream substrate JNK and shear its downstream substrate XBP1 mRNA (Li X et al. 2015; Shi et al. 2018). The unspliced XBP-1 (XBP1u) protein is translated from XBP1mRNA without splicing, while being spliced a 26 BP intron, XBP1u transforms into XBP1s which is an active form of XBP1 and has strong transcriptional activity (Li X et al. 2015). XBP1s, which can enter the nucleus and bind to the X-box in the promoter sequence of unfolded protein response element (UPRE), increase the expression of its target genes such as ER degrading enhancer alpha-mannosidase-like protein (EDEM) and GRP78 which promote the degradation of unfolded proteins, and thus maintains the homeostasis of ER and plays an anti-apoptotic role (Yoshida et al. 2001).

Besides, severe ER stress activates JNK and CHOP-mediated apoptosis (Kato et al. 2012; Li Y et al. 2015). Interestingly, XBP1s can also activate NLRP3-mediated inflammatory response (Yue et al. 2016). NLRP3 is a member of the NLRs receptor family. NLRP3 inflammasome is a complex of NLRP3, pro-caspase-1 and ASC. When cells are stimulated, the junction protein ASC recruits pro-caspase-1, which self-secures to form the enzyme-active caspase-1, and the activated caspase-1 secures the pro-IL-1β, transforming it into mature IL-1β, which is secreted into extracellular medium to trigger an inflammatory response (Yao et al. 2017).

We found that the mRNA and protein expression of XBP1u, XBP1s, NLRP3, caspase-1 as well as the ratio of p-IRE1 α/IRE1α, and protein expression of IL-1β in the OGD/R group and IRE1α overexpression group were significantly increased which is consistent with the above reports. These results suggest that IRE1α/XBP1 signalling pathway plays an important role in the expression of IL-1β after cerebral I/R, ICA can significantly reduce the expression of the above factors in OGD/R group and overexpression of IRE1α significantly reverse the effects of ICA, suggesting that ICA can reduce the expression of NLRP3 and caspase-1 and the secretion of IL-1β by inhibiting the IRE1α/XBP1 signalling pathway.

It is reported that up-regulated TNF-α and IL-6 was associated with higher XBP1s at both the mRNA and protein level, and inhibition of IRE1α‐dependent XBP1s decreased LPS-up-regulated TNF-α and IL-6 at both the mRNA and protein level, indicating that up-regulation of XBP1s mediates TNF-α and IL-6 production (Lubamba et al. 2015). It is also reported that silencing NLRP3 gene or inhibiting caspase-1 significantly reduced the expression of IL-1β, IL-6 and TNF-α proteins in microglia, suggesting that NLRP3 and caspase-1 regulates the expression of IL-1β, IL-6 and TNF-α proteins (Yang et al. 2015). However, the molecular mechanism underlying regulation of IL-6 and TNF-α expression by IRE1α-XBP1 signalling pathway is not fully understood. In this study, the results showed that compared with the OGD/R group, the expression of IL-6 and TNF-α was significantly increased in IRE1 overexpression group, which was consistent with the literature. ICA could significantly reduce the expression of the above factors in OGD/R group (*p* < 0.05 and *p* < 0.01) and overexpression of IRE1 significantly reverse the effects of ICA (*p* < 0.01). Based on the above results and literature, it is suggested that ICA could reduce the expression of IL-1β, IL-6 and TNF-α by regulating the IRE1/XBP1s pathway. As inflammatory response is one of the important factors causing brain injury after stroke, the anti-inflammatory effect of ICA may provide a potential therapeutic strategy for the treatment of brain injury after stroke.

## Conclusions

The present study has demonstrated that ICA suppresses inflammation induced by OGD/R via IRE1/XBP1s pathway. ICA is a promising candidate drug for inhibiting inflammatory response after cerebral I/R.
